# Physical activity and sport-specific training patterns in Swedish sporting and working trial dogs—A questionnaire survey

**DOI:** 10.3389/fvets.2022.976000

**Published:** 2022-11-01

**Authors:** Ann Essner, Amie L. Hesbach, Helena Igelström, Catarina Kjellerstedt, Kristina Svensson, Helga Westerlind

**Affiliations:** ^1^Djurkliniken Gefle, IVC Evidensia, Gävle, Sweden; ^2^Department of Women's and Children's Health, Uppsala University, Uppsala, Sweden; ^3^EmpowerPhysio, The Hague, Netherlands; ^4^Veterinär Catarina Kjellerstedt, Vallentuna, Sweden; ^5^Tolleruds Gård 116, Karlstad, Sweden; ^6^Clinical Epidemiology Unit, Department of Medicine Solna, Karolinska Institutet, Stockholm, Sweden

**Keywords:** physical conditioning, physical activity, sports medicine, sporting dogs, sport specialization, surface, warm-up, working dogs

## Abstract

**Objective:**

To explore physical activity patterns, including conditioning exercise and sport-specific training, and management routines utilized by handlers of Swedish sporting and working dogs participating in agility, obedience, rally obedience and working trial disciplines.

**Procedures:**

Dog handlers provided information on competition-level dogs through an internet-based cross-sectional and descriptive survey on physical activity, sport-specific training and management. Results are reported overall and stratified by participation in specific disciplines.

**Results:**

We received 1615 replies to the questionnaire. After data cleaning, 1582 dogs (98%) remained for the analysis. Of these, 430 participated in agility, 790 in obedience, 596 in rally obedience, and 847 dogs had competed in a working trial, i.e., messenger, protection, search or tracking. Number of disciplines performed by each dog varied between one and five. Most common was participation in one (*n* = 767, 48%) or two (*n* = 541, 34%) disciplines. Out of the dogs competing in one discipline, 38% (*n* = 294) were considered to be specialized as they actively trained only that discipline for ≥10 months per year. The vast majority of the dogs (*n* = 1129, 71%) received more than 1 h of daily physical activity, e.g., walks, and only *n* = 51 (3%) were never exercised off-leash. Preferred self-selected gait was trot (*n* = 907, 57%) and gallop (*n* = 499, 32%). A fifth (*n* = 319, 20%) never played with other dogs. The majority (*n* = 1328, 84%) received more than 1 h of vigorous physical conditioning exercise per week. Almost three quarters (*n* = 1119, 71%) participated in physical conditioning exercise. Two thirds (*n* = 953, 60%) participated in at least 3 h of sport-specific training per week and only a very small portion (*n* = 35, 2%) trained their specific discipline less than once per week. Median total work load, i.e., all daily physical activity, vigorous physical conditioning exercise and sport-specific training, was 16.5 h per week.

**Conclusion and clinical relevance:**

We observe physical activity at moderate to high durations and moderate to vigorous intensities among Swedish sporting and working trial dogs. Most dogs received physical conditioning exercise, but not all dogs were warmed up before training and competition. Our study provides veterinary professionals and dog trainers with valuable insights on the physical exposures and management routines of sporting and working trial dogs.

## Introduction

Besides offering companionship, dog sports are popular activities with dogs (1). Furthermore, dog ownership is associated with increased general physical activity in humans (2). Human participants in dog sports have varying backgrounds and purposes, from casual leisure to occupational devotion, and participation is at local to international level (3). Sporting and working trials are becoming popular parts of the canine industry and veterinary professional are regularly treating these dogs in clinic. Therefore, understanding the work load and demands requested from canine athletes is essential for being an effective veterinary professional (4–6). In dog sports, handlers navigate the dogs through physically and mentally demanding tasks, e.g., heelwork, obstacles to overcome, searching for people in the forest and objects to retrieve (4). The physical requirements vary among disciplines, where disciplines such as agility and protection involve tasks that require muscle strength and power, while rally obedience has lower physical impact (4, 7, 8). Other disciplines, like searching and tracking people demand cardiorespiratory and muscular endurance from the dog (9–11).

When participating in sports and working trials, dogs need to be prepared for sport-specific tasks (12–14). Physical fitness, as well as sport and field specific training, are thus integral parts of performance in trials and competitions. Physical fitness includes cardiorespiratory (i.e., aerobic and anaerobic capacity) and neuromuscular (i.e., muscle strength, mobility, balance) components. Body composition (i.e., lean and fat body mass) and nutritional prerequisites, need to be optimized for the dog to reach full athletic performance (15–17).

In the fields of rehabilitation and physical education and training, physical activity and physical conditioning exercises are described according to the “F.I.T.T.”-principle (i.e., frequency, intensity, time (duration), and type of exercise) to facilitate exercise prescription (18, 19). Current definitions of canine physical activity are vague. In human literature, physical activity has been recognized as any bodily movement produced by the contraction of skeletal muscles that results in an increase in caloric expenditure over resting energy requirement (19, 20). Physical conditioning exercise is done to improve and/or maintain physical fitness components, and is per definition a type of physical activity consisting of planned, structured, and repetitive bodily movement (13, 15, 19, 20). Sport-specific training in dogs is another type of conditioning, an associative learning process, with regards to learning relevant tasks related to a sport or working discipline (21).

Understanding of how dogs' physical activity patterns are related to their health, well-being, injury and disease in various life stages and performance is increasingly important to canine welfare (22). However, science-based guidelines for achieving health and sport-specific benefits from physical activity in dogs are still sparse. A unified way of reporting physical activity in dogs is essential to facilitate future studies on relationships to canine health and well-being.

Consensus with regards to how to define physical activity patterns in dogs has yet to be established.

The lack of a definition may lead to different procedures to capture and express physical activity in dogs, and even more specifically in working and performing canine athletes. Physical activity pattern can be defined as a way in which physical activities, including physical conditioning exercise, and periodization of sport-specific training are repeated over time (20, 23, 24). A few studies have investigated canine physical activity at various intensities or according to duration (25–28). Durations of physical activity at various intensities have been assessed in privately owned free-ranging dogs, farm dogs, and family dogs by measurements recorded from an accelerometer device (25). Intensity and time-related categories based on canine gaits and duration per day have been described in previous studies. With regards to gait, one study defined walk as light exercise and trot and faster gaits as moderate to vigorous exercise (26), and a second study defined slow walk on a lead as light to moderate activity, and running off leash as vigorous physical activity (27). With regards to time, three time-related categories have been used to describe daily duration of physical activity, i.e., <1 h per day (“low”), 1–3 h per day (“moderate”), and >3 h per day (“high”) (29–31).

Although there are several questionnaires validated to measure and monitor levels of physical activity in humans (32), there are no validated owner-reported instruments to capture physical activity and sedentary behaviors in various dog populations. Dog owner-reported measures of physical activity may provide important contextual information (28, 33–40). Hence, estimations of physical activity patterns may be based on subjective means (32, 41), e.g., owner-reported questionnaires (28), owner-reported logs and direct observation, and/or be based on measurements from an objective device, e.g., accelerometer (42, 43), pedometer (44–46), heart rate monitor (47) or smart devices (48, 49).

Recently, several studies have been published on daily physical activity and sport-specific training parameters and their associations to injury in agility dogs (33–35, 39, 40, 50–54). There is however a lack of research focusing on physical activity patterns in sporting and working trial dogs from other disciplines. Canine sporting competitions and working dog trials have been organized by the Swedish Working Dog Association (SWDA) since 1918 (55, 56). The SWDA is a non-profit members' association organized under the Fédération Cynologique Internationale (FCI), the world governing body for canine sporting disciplines (57). The implementation of sporting and working trial disciplines are similar in many countries and international competitions under the same rules are applied, e.g., in obedience classes.

The objective of this study was to explore physical activity patterns, including physical conditioning exercise and sport-specific training, and management routines among sporting and working trial dogs participating in various disciplines in Sweden.

## Materials and methods

### Dogs and data collection

This research was an online survey with a cross-sectional and descriptive study design. Data were collected for eligible dogs via a questionnaire distributed electronically to handlers of dogs competing in agility, obedience, rally obedience, mondioring, working trials (i.e., messenger, patrol, protection, search, tracking, International Utility Dog trials, International Nordics Style and BH-VT exams) organized by the Swedish Working Dog Association (SWDA) (55, 58).

Participation in the internet-based questionnaire was not restricted to geographic location or type of dog. Inclusion criteria were: dog born 2005 or later; participating in a sport discipline and/or working trial organized by the SWDA at any level at least once; owner access to the internet; and willingness and ability to complete an online survey in Swedish. Participation was initiated when the respondent clicked an embedded hyperlink that directly accessed the appropriate survey. The respondent could fill in the questionnaire for several additional dogs.

### Questionnaire

An online survey was developed by veterinarians, veterinary physiotherapists, a statistician, and experienced obedience and working trial judges. The questionnaire was tested in a pilot group of dog handlers and adjusted accordingly prior to publishing. The final version of the questionnaire contained mainly close-ended multiple choice questions in Swedish. The results from a qualitative content analysis of narrative data from open-ended questions in the survey have been published elsewhere (59). The survey was distributed by means of an internet survey site (Google Forms, Google LLC, Mountain View, CA, USA) to facilitate data collection. The survey was initiated on February 1, 2019 and remained open for 2 months until April 3, 2019. Recruitment strategies included advertisements with the survey link at the internet sites of the SWDA (02/01/2019 and 03/25/2019) and the Swedish Kennel Club (03/27/2019), and social media groups. Survey participation, i.e., responding to an anonymous online questionnaire, was entirely voluntary.

Based on the F.I.T.T.-principle, items in the questionnaire were targeting various components of physical activity (15, 18, 19). Frequencies, two levels of intensity, time (duration) and types of physical activities and sport-specific training were reported by respondents. Duration of physical activity was divided into low-moderate and vigorous level of intensity, respectively (27, 41, 60). Low to moderate intensity was represented by one item targeting daily physical activity, e.g., walks, and vigorous intensity was targeted by physical activities resulting in hard panting, e.g., off-leach, running, swimming (26, 27, 41, 60). In addition, the questionnaire also included items about the dogs' characteristics, such as age, weight, sex, breed, health history, and management routines, e.g., surfaces used for physical activity and sport-specific training, frequency and type of warm-up activity. In questions concerning types of physical activity, surfaces used, and types of warm-up activities the respondents were given an opportunity to add information in open-field boxes. Table 1 shows the details of the topics and variables relevant for this study.

**Table 1:** Questions and variables regarding demographics, health history, physical activity, sport-specific training, and management.

**Topic**	**Variable**	**Categories**
Individual characteristics	Age	<1 year/1–2 years/2–4 years/4–6 years/6–8 years/8–10 years/>10 years/Deceased
	Sex	Sexually intact male/Neutered male/Sexually intact female/Spayed female
	Weight	Kilograms
	Breed group	Breed group 1–10 by Federation Cynologique International
	Breed	Breed by Federation Cynologique International or breed acknowledged by the Swedish Kennel Club/mixed breed
Health history	Hip dysplasia (Federation Cynologique International grade)	Grade A-E/do not know
	Elbow dysplasia (Federation Cynologique International grade)	No remarks/minor/moderate/severe/do not know
	Mental evaluation	Participated in official mental test/mental description/dog behavior personality description yes/no
	Injury	Never/Once/2–3 times/4 times or more
Physical activity	Time (duration) of low to moderate daily physical activity (minutes per day)	<15 min/15–30 min/30–60 min/1–2 h/2–3 h/3–4 h/>5 h
	Time (duration) of vigorous physical conditioning exercise (e.g., off-leach, running, swimming) (minutes per week)	0 min/ <30 min/30–60 min/60–90 min/90–120 min/120–180 min/>180 min
	Proportion of time spent off-leash	Never/ <25%/25–50%/50–75%/75–100%
	Preferred self-selected gait	Unwilling to move/Walk/Pace/Trot/Gallop/Do not know
	Physical conditioning exercise	Yes/no
	Content of physical conditioning exercise	Categories defined according to targeted component of canine fitness i.e., cardiorespiratory endurance, musculoskeletal components or a combination of both.
	Frequency of play sessions with other dogs (monthly)	Never/Approximately once per month/Approximately once every second week/Approximately once per week/Several times per week/Every day/Several times per day
	Surface used for physical activity	Natural grass/Turf/Forest/Field/Gravel/Sand/Asphalt/Stone/Concrete/Snow/Ice/Indoor venue/Home flooring/Other—water/Other—mobile/Other—soft. (see Supplementary Table 2).
Sport-specific training	Time (duration)—hours per week in categories and mean per category	0–1 h/1–2 h/2–3 h/3–5 h/5–7 h/7–10 h/>10 h
	Frequency of sport-specific training over a month	Never/Once a month/Every other week/Once per week/Several times per week/Daily/Several times per day
	Frequency of selected types of activities over the year	Never/Once a month/Every other week/Once per week/Several times per week/Daily/Several times per day. Reported as frequencies and total number of physical activities.
		Selected activities: tracking, search, mushing, obedience, messenger, International Utility Dog trial phase c - protection, Swedish schutzhund, mondioring, protection related to Police K9 or guard dog duty, hunting, game tracking, search and rescue, freestyle, patrol, racing, herding, agility, nose work, rally obedience, drag weight/weight pull.
	Participation in dog sports and working trials	Participation in agility, obedience, rally, any working dog discipline, messenger, protection, search, tracking, and number of disciplines
	Sport specialization - sport training and competition in one Swedish Working Dog Association discipline for ≥10 months per year.	Presented as a proportion by sport discipline and by the whole cohort.
		Specializing in agility, obedience, rally obedience, working trial discipline (i.e., messenger, protection, search, tracking).
	Surface used for sport-specific training	Natural grass/Turf/Forest/Field/Gravel/Sand/Asphalt/Stone/Concrete/Snow/Ice/Indoor venue/Home flooring/Other—water/Other—mobile/Other—soft. (see Supplementary Table 2).
Total work load	Time (duration) in low to moderate daily physical activity, vigorous physical conditioning exercise and sport-specific training (hours per week in categories and mean per category)	Median (inter quartile range)
Warm-up	Frequency of participation	Never/Seldom/Sometimes/Often/Always
	Time (duration) (minutes per session)	0 min/1–10 min/11–20 min/21–30 min/31 min or more
	Content of warm-up activities	Categories defined according to targeted effect general/sport specific/mobility/passive/other

### Data analysis and statistical methods

Descriptive baseline characteristics were summarized using frequencies and proportion (%) in categorical data and for continuous data distributions were manually inspected. Mean and standard deviation (SD) was calculated for normally distributed variables and median and inter quartile range (IQR) for non-normally distributed data. Variables regarding demographics, health history, physical activity, sport-specific training and management are described in Table 1.

Working trial disciplines including mondioring were further combined into four categories, i.e., messenger, protection, search, tracking. These are defined in detail in Supplementary Table 1. We further calculated the total number of disciplines a dog participated in.

Sport specialization was defined as competing in only one discipline and training that sport for ≥ 10 months per year. This is analogous with sport specialization in human adolescents (23, 61).

Total work load per week was calculated as assigning the middle of time point in the interval for duration of daily physical activity, duration of vigorous physical conditioning exercise, and duration of sport-specific training, per week. Thus, an interval of 0–1 h yielded a training time of 30 min, 1–2 h was set to 90 min, and so on, except for the daily physical activity category of <15 min per day, which was assigned 0 min. The mean total work hours per week was calculated as [(duration of daily physical activity per day × 7) + duration of vigorous physical conditioning exercise + duration of sport-specific training per week]/60.

Physical conditioning exercise to improve or maintain canine fitness, was categorized as “cardiorespiratory”, “musculoskeletal”, or a “combination” of both (4, 13, 15, 19, 20). Cardiorespiratory activities included aerobic and/or anaerobic endurance, e.g., intervals in gallop, galloping in sand, trot or gallop off-leash with handler riding bike. Musculoskeletal activities included muscular endurance, strength, power, stability, balance, mobility or agility, such as parkour, drag weight, weight vest during walking, jumping technique exercises, balance training exercises, tricks, static stretching, walking in snow and on uneven surfaces, underwater treadmill training, or cavaletti. Activities requiring both cardiorespiratory and musculoskeletal components of physical fitness, e.g., hill climbing, running, agility, swimming, canicross, bikejioring, off-leash exercise in the forest, treadmill, were categorized as a “combination”.

Warm-up activities were categorized as “general”, “sport specific”, “mobility”, “passive” and “other” analogous with components previously described in human and canine literature (15, 22, 24, 62, 63). General warm-up activities aiming at increasing body temperature included locomotion in walk and/or trot. Sport-specific warm-up activities included movements and tasks that were to be performed in the upcoming discipline, e.g., heelwork, jumping, bite work, off-leash search for objects, intervals in gallop. Mobility warm-up activities included dynamic and/or static stretching with a purpose to increase flexibility, e.g., play, tricks, walking in circles or with increased active joint range of motion, locomotion off-leash, short intervals in canter. Passive warm-up included massage and/or warm blanket, and “Other” warm-up included unspecified physical warm-up and/or mental preparation.

Main surfaces used for physical activity, physical conditioning exercise and sport-specific training were categorized into 15 different categories, specified in Supplementary Table 2.

Sensitivity analyses were performed to assess the possible influence of recall bias on inconsistencies in reported frequency of sport-specific training over the year. In the first sensitivity analysis we excluded all dogs that did not participate in sport-specific training during the past year. A second sensitivity analysis was performed in which all deceased dogs were excluded.

### Ethical consideration

This research was conducted as online reported data from handlers of sporting and working trial dogs, without subjecting the dogs to any kind of stress or suffering. The respondents were informed and free to choose whether to participate in the study. All respondents were debriefed in writing about the content and purpose of the study. In the first paragraph of the online questionnaire it was stated that by completing and submitting the online questionnaire the respondents were providing their informed consent. No personal or sensitive data were collected from the respondents and all data on sporting and working dogs were anonymous. Respondents could possibly withdraw their responses only by contacting the responsible researcher (A.E.) with descriptive data about their dog. Otherwise, the responses could not be traced back to detect individual responses.

## Results

### Cohort characteristics

A total of 1615 survey answers were received. Out of these, 29 were excluded due to incomplete responses on sport participation. Four dogs were further excluded due to inconsistencies between reported participation in dog training activities and reported training time, resulting in 1582 unique dogs included in the analysis. Full characteristics are presented in Table 2. The age category 4–6 years was the most common (*n* = 428, 27.1%). Of all dogs, *n* = 692 (44%) were intact females, *n* = 205 (13%) spayed females, *n* = 518 (33%) intact males and *n* = 167 (11%) neutered males. The median weight was 23 kg (IQR 14 kg, *n* missing = 6). Most dogs were from FCI group 1 (Sheepdogs and Cattle dogs) (*n* = 895, 57%), while group 8 (Retrievers, Flushing dogs, Water dogs) and group 2 (Pinscher and Schnauzer—Molossoid and Swiss Mountain and Cattle dogs) were second and third most common (*n* = 232 (15%) and *n* = 200 (13%), respectively). The distribution across the FCI breed groups can be seen in Table 2. The five most common breeds were German Shepherd Dog (*n* = 205, 13%), Border Collie (*n* = 133, 8%), Belgian Shepherd, Malinois (*n* = 111, 7%), Australian Shepherd (*n* = 86, 5%) and Australian Kelpie (*n* = 76, 5%). See Supplementary Table 3 for the distribution across all breeds. Only a small proportion of the dogs did not have an FCI evaluation for hip dysplasia (*n* = 256, 16%) or elbow dysplasia (*n* = 418, 26%), but *n* = 189 (12%) of the dogs were reported to have hip dysplasia and *n* = 65 (4%) elbow dysplasia (Table 2). Moreover, *n* = 1229 (78%) had participated in any of the official mental evaluations available in Sweden and *n* = 844 (53%) had participated in structural conformation evaluation performed by an official judge, e.g., at open dog show.

**Table 2:** Demographics and characteristics of sporting and working trial dogs (*n* = 1582).

	**Full cohort**	**Agility**	**Obedience**	**Rally** **obedience**	Working^*^
N dogs	1582	430	790	596	847
**Age**					
<1 year	2 (0.1)	0 (0)	1 (0.1)	1 (0.2)	0 (0)
1–2 years	91 (5.8)	12 (2.8)	48 (6.1)	35 (5.9)	26 (3.1)
2–4 years	387 (24.5)	99 (23)	190 (24.1)	117 (19.6)	200 (23.6)
4–6 years	428 (27.1)	122 (28.4)	204 (25.8)	173 (29)	216 (25.5)
6–8 years	268 (16.9)	77 (17.9)	136 (17.2)	104 (17.4)	153 (18.1)
8–10 years	207 (13.1)	62 (14.4)	110 (13.9)	95 (15.9)	120 (14.2)
>10 years	94 (5.9)	43 (10)	50 (6.3)	45 (7.6)	51 (6)
Deceased	105 (6.6)	15 (3.5)	51 (6.5)	26 (4.4)	81 (9.6)
**Gender**					
Sexually intact male	518 (32.7)	119 (27.7)	257 (32.5)	174 (29.2)	307 (36.2)
Neutered male	167 (10.6)	59 (13.7)	81 (10.3)	68 (11.4)	69 (8.1)
Sexually intact female	692 (43.7)	180 (41.9)	359 (45.4)	267 (44.8)	361 (42.6)
Spayed female	205 (13)	72 (16.7)	93 (11.8)	87 (14.6)	110 (13)
**Median weight (kgs) (IQR)**	23 (14)	14 (10.7)	24 (12)	20 (13)	28 (11)
**FCI Breed group**					
1 Sheepdogs and Cattledogs	895 (56.6)	237 (55.1)	460 (58.2)	266 (44.6)	564 (66.6)
2 Pinscher and Schnauzer	200 (12.6)	19 (4.4)	102 (12.9)	47 (7.9)	152 (17.9)
3 Terriers	59 (3.7)	29 (6.7)	27 (3.4)	34 (5.7)	14 (1.7)
4 Dachshunds	2 (0.1)	2 (0.5)	0 (0)	0 (0)	0 (0)
5 Spitz and primitive types	44 (2.8)	21 (4.9)	12 (1.5)	32 (5.4)	7 (0.8)
6 Scent hounds and related breeds	7 (0.4)	1 (0.2)	3 (0.4)	6 (1)	1 (0.1)
7 Pointing Dogs	16 (1)	3 (0.7)	8 (1)	12 (2)	3 (0.4)
8 Retrievers/Flushing and Water Dogs	232 (14.7)	39 (9.1)	137 (17.3)	123 (20.6)	94 (11.1)
9 Companion and Toy Dogs	72 (4.6)	49 (11.4)	26 (3.3)	41 (6.9)	10 (1.2)
10 Sighthounds	9 (0.6)	3 (0.7)	1 (0.1)	9 (1.5)	1 (0.1)
Mixed breed	46 (2.9)	27 (6.3)	14 (1.8)	26 (4.4)	1 (0.1)
**Hip dysplasia (FCI grade)**					
A	776 (49.1)	164 (38.1)	434 (54.9)	269 (45.1)	498 (58.8)
B	362 (22.9)	88 (20.5)	178 (22.5)	124 (20.8)	211 (24.9)
C	157 (9.9)	36 (8.4)	83 (10.5)	62 (10.4)	86 (10.2)
D	30 (1.9)	8 (1.9)	15 (1.9)	10 (1.7)	17 (2)
E	2 (0.1)	0 (0)	1 (0.1)	0 (0)	2 (0.2)
Unknown	255 (16.1)	134 (31.2)	79 (10)	131 (22)	33 (3.9)
**Elbow dysplasia (FCI grade)**					
Normal	1099 (69.5)	214 (49.8)	596 (75.4)	378 (63.4)	704 (83.1)
Mild	51 (3.2)	5 (1.2)	30 (3.8)	18 (3)	35 (4.1)
Moderate	8 (0.5)	1 (0.2)	6 (0.8)	4 (0.7)	5 (0.6)
Severe	6 (0.4)	0 (0)	4 (0.5)	1 (0.2)	5 (0.6)
Unknown	418 (26.4)	210 (48.8)	154 (19.5)	195 (32.7)	98 (11.6)
**Official mental evaluation**	1229 (77.7)	217 (50.5)	673 (85.2)	423 (71)	842 (99.4)
**Official conformational evaluation**	844 (53.4)	212 (49.3)	432 (54.7)	376 (63.1)	476 (56.2)

Data are presented in frequencies and proportions (%).

* Working trial disciplines were defined as Swedish Schutzhund, tracking (SWDA), search (SWDA), messenger (SWDA), patrol (SWDA), International Utility Dog trials (tracking, obedience, protection, search and rescue), International Nordic Style, BH/VT, and mondioring. SWDA, Swedish Working Dog Association.

The year prior to the study, *n* = 1329 (84%) of the dogs, had been trained for competition. Over half of the dogs (*n* = 919, 58%) had ever suffered from an injury. The proportion of injured dogs varied slightly across the disciplines with the highest proportion in agility dogs (*n* = 276, 64%) and the lowest in obedience (*n* = 441, 56%).

### Physical activity patterns

Almost one third of the dogs (*n* = 453, 29%) received <1 h per day of physical activity, e.g., walks, and only 3% (*n* = 51) were never exercised off leash. Trot was reported as the primary self-selected gait in 57% (*n* = 907) of the dogs and gallop in 32% (*n* = 499). A fifth (*n* = 319, 20%) of the dogs never played with other dogs. The majority of the dogs (*n* = 1328, 84%) had more than 1 h of vigorous physical exercise per week (Table 3).

**Table 3:** Physical activity including physical conditioning exercise reported in the full cohort of competition dogs (*n* = 1582) and stratified by participation in various disciplines.

	**Full cohort**	**Agility**	**Obedience**	**Rally** **obedience**	Working^*^
N dogs	1582	430	790	596	847
**Daily physical activity (e.g., walks)**					
<15 min	12 (0.8)	2 (0.5)	4 (0.5)	2 (0.3)	9 (1.1)
15–30 min	82 (5.2)	19 (4.4)	41 (5.2)	25 (4.2)	48 (5.7)
30–60 min	359 (22.7)	98 (22.8)	182 (23.0)	138 (23.2)	201 (23.7)
1–2 h	724 (45.8)	203 (47.2)	357 (45.2)	272 (45.6)	386 (45.6)
2–3 h	321 (20.3)	86 (20.0)	158 (20.0)	129 (21.6)	158 (18.7)
3–4 h	64 (4.0)	16 (3.7)	40 (5.1)	23 (3.9)	35 (4.1)
>5 h	20 (1.3)	6 (1.4)	8 (1.0)	7 (1.2)	10 (1.2)
**Duration (time) of vigorous physical conditioning exercise (e.g., off-leash, running, swimming) per week**					
0 min	8 (0.5)	3 (0.7)	5 (0.6)	3 (0.5)	4 (0.5)
<30 min	76 (4.8)	23 (5.3)	41 (5.2)	49 (8.2)	28 (3.3)
30–60 min	170 (10.7)	43 (10.0)	92 (11.6)	79 (13.3)	93 (11.0)
1–1.5 h	240 (15.2)	78 (18.1)	114 (14.4)	99 (16.6)	121 (14.3)
1.5–2 h	244 (15.4)	68 (15.8)	120 (15.2)	81 (13.6)	126 (14.9)
2–3 h	292 (18.5)	79 (18.4)	146 (18.5)	105 (17.6)	158 (18.7)
>3 h	552 (34.9)	136 (31.6)	272 (34.4)	180 (30.2)	317 (37.4)
**Percentage of physical activity spent off leash**					
Never	51 (3.2)	18 (4.2)	22 (2.8)	28 (4.7)	21 (2.5)
<25%	257 (16.2)	86 (20.0)	126 (15.9)	116 (19.5)	111 (13.1)
25–50%	265 (16.8)	82 (19.1)	131 (16.6)	119 (20.0)	133 (15.7)
50–75%	369 (23.3)	100 (23.3)	203 (25.7)	128 (21.5)	199 (23.5)
75–100%	640 (40.5)	144 (33.5)	308 (39.0)	205 (34.4)	383 (45.2)
**Frequency of play sessions with other dogs**					
Never	319 (20.2)	56 (13.0)	159 (20.1)	85 (14.3)	225 (26.6)
Monthly	191 (12.1)	43 (10.0)	110 (13.9)	73 (12.2)	105 (12.4)
Every other week	97 (6.1)	28 (6.5)	51 (6.5)	40 (6.7)	50 (5.9)
Weekly	131 (8.3)	39 (9.1)	74 (9.4)	60 (10.1)	67 (7.9)
Several times per week	200 (12.6)	58 (13.5)	98 (12.4)	91 (15.3)	96 (11.3)
Daily	392 (24.8)	129 (30.0)	197 (24.9)	153 (25.7)	188 (22.2)
Several times per day	252 (15.9)	77 (17.9)	101 (12.8)	94 (15.8)	116 (13.7)
**Preferred self-selected gait**					
Unwilling to move	1 (0.1)	0 (0.0)	0 (0.0)	1 (0.2)	0 (0.0)
Walk	41 (2.6)	14 (3.3)	20 (2.5)	23 (3.9)	17 (2.0)
Pace	120 (7.6)	38 (8.8)	74 (9.4)	50 (8.4)	73 (8.6)
Trot	907 (57.3)	249 (57.9)	443 (56.1)	359 (60.2)	478 (56.4)
Gallop	499 (31.5)	121 (28.1)	248 (31.4)	158 (26.5)	274 (32.3)
I don't know	14 (0.9)	8 (1.9)	5 (0.6)	5 (0.8)	5 (0.6)

Data are presented in frequencies and proportions (%).

* Working disciplines were defined as Swedish Schutzhund, tracking (SWDA), search (SWDA), messenger (SWDA), patrol (SWDA), International Utility Dog trials (tracking, obedience, protection, search and rescue), International Nordic Style, BH/VT, and mondioring. SWDA, Swedish Working Dog Association.

Almost three quarters of the participants (*n* = 1119, 71%) added physical conditioning exercise to improve and/or maintain their dogs' physical capacity in sports. Nearly half of the participants (*n* = 732, 46%) were addressing musculoskeletal components of physical capacity through physical conditioning activities (Tables 4, 5).

**Table 4:** Sport-specific training and physical conditioning exercise in the full cohort of competition dogs (*n* = 1582) and stratified by participation in various disciplines.

	**Full cohort**	**Agility**	**Obedience**	**Rally obedience**	Working^*^
N dogs	1582	430	790	596	847
**Duration of sport-specific training per week**					
0–1 h	103 (6.5)	46 (10.7)	42 (5.3)	39 (6.5)	35 (4.1)
1–2 h	238 (15.0)	85 (19.8)	102 (12.9)	100 (16.8)	101 (11.9)
2–3 h	288 (18.2)	92 (21.4)	142 (18.0)	135 (22.7)	146 (17.2)
3–5 h	398 (25.2)	116 (27.0)	208 (26.3)	150 (25.2)	202 (23.8)
5–7 h	303 (19.2)	56 (13.0)	166 (21.0)	105 (17.6)	179 (21.1)
7–10 h	153 (9.7)	21 (4.9)	77 (9.7)	46 (7.7)	107 (12.6)
More than 10 h	99 (6.3)	14 (3.3)	53 (6.7)	21 (3.5)	77 (9.1)
**Frequency of sport-specific training in total**					
Never	1 (0.1)	0 (0.0)	1 (0.1)	0 (0.0)	1 (0.1)
Once a month	8 (0.5)	4 (0.9)	3 (0.4)	4 (0.7)	1 (0.1)
Every other week	26 (1.6)	12 (2.8)	9 (1.1)	15 (2.5)	6 (0.7)
Once per week	123 (7.8)	45 (10.5)	56 (7.1)	49 (8.2)	63 (7.4)
Several times per week	946 (59.8)	272 (63.3)	466 (59.0)	352 (59.1)	505 (59.6)
Daily	426 (26.9)	88 (20.5)	217 (27.5)	154 (25.8)	250 (29.5)
Several times per day	52 (3.3)	9 (2.1)	38 (4.8)	22 (3.7)	21 (2.5)
**Total work load per week** ^**^					
Median (IQR)	16.5 (9.0)	15.5 (8.6)	17.0 (9.6)	15.8 (9.2)	16.8 (9.4)
Content of physical conditioning exercise^***^					
Cardiorespiratory^1^	79 (5.0)	9 (2.1)	40 (5.1)	26 (4.4)	53 (6.3)
Muscular^2^	732 (46.3)	175 (40.7)	376 (47.6)	250 (41.9)	440 (51.9)
Combination^3^	308 (19.5)	133 (30.9)	145 (18.4)	138 (23.2)	109 (12.9)

Data are presented in frequencies and proportions (%).

* Working trial disciplines were defined as Swedish Schutzhund, tracking (SWDA), search (SWDA), messenger (SWDA), patrol (SWDA), International Utility Dog trials (tracking, obedience, protection, search and rescue), International Nordic Style, BH/VT, and mondioring. SWDA, Swedish Working Dog Association.

** Total work load per week was defined as hours per week in daily physical activity, vigorous physical conditioning exercise and sport-specific training.

*** Conditioning was defined as physical exercises target to improve and/or maintain cardiorespiratory or musculoskeletal physical fitness components, or as a combination of both.

1 Activities including aerobic and/or anaerobic endurance, e.g., intervals in gallop, galloping in sand, trot or gallop of leash with handler riding bike.

2 Activities requiring muscular endurance, strength, power, stability, balance, mobility or agility, such as parkour, drag weight, weight vest during walking, jumping technique exercises, balance training exercises, tricks, static stretching, walking in snow and on uneven surfaces, under water treadmill training, cavaletti.

3 Activities requiring both cardiorespiratory and musculoskeletal components of physical fitness, e.g., hill climbing, running, agility, swimming, canicross, bikejioring, off-leash exercise in the forest, treadmill.

**Table 5:** Sport-specific training and physical conditioning exercise stratified by working trial disciplines.

	**Messenger**	**Protection**	**Search**	**Tracking**
N dogs	33	169	226	667
**Duration of sport-specific training per week**				
0–1 h	0 (0.0)	4 (2.4)	9 (4.0)	27 (4.0)
1–2 h	4 (12.1)	19 (11.2)	28 (12.4)	83 (12.4)
2–3 h	5 (15.2)	16 (9.5)	39 (17.3)	113 (16.9)
3–5 h	7 (21.2)	41 (24.3)	56 (24.8)	150 (22.5)
5–7 h	4 (12.1)	50 (29.6)	47 (20.8)	151 (22.6)
7–10 h	11 (33.3)	24 (14.2)	25 (11.1)	84 (12.6)
>10 h	2 (6.1)	15 (8.9)	22 (9.7)	59 (8.8)
**Frequency of sport-specific training**				
Never	0 (0.0)	0 (0.0)	0 (0.0)	1 (0.1)
Once a month	0 (0.0)	0 (0.0)	1 (0.4)	1 (0.1)
Every other week	0 (0.0.)	0 (0.0)	1 (0.4)	6 (0.9)
Once per week	3 (9.1)	8 (4.7)	17 (7.5)	49 (7.3)
Several times per week	17 (51.5)	102 (60.4)	142 (62.8)	400 (60.0)
Daily	13 (39.4)	57 (33.7)	58 (25.7)	193 (28.9)
Several times per day	0 (0.0)	2 (1.2)	7 (3.1)	17 (2.5)
Total work load per week^*^				
Median (IQR)	20.8 (9.8)	17.0 (7.2)	17.5 (10.0)	17.0 (9.5)
Content of physical conditioning exercise^**^				
Cardiorespiratory^1^	5 (15.2)	12 (7.1)	13 (5.8)	42 (6.3)
Muscular^2^	21 (63.6)	102 (60.4)	123 (54.4)	339 (50.8)
Combination^3^	3 (9.1)	19 (11.2)	24 (10.6)	84 (12.6)

Data are presented in frequencies and proportions (%).

* Total work load per week was defined as hours per week in daily physical activity, vigorous exercise and sport-specific training.

** Conditioning was defined as physical conditioning exercises target to improve and/or maintain cardiorespiratory or musculoskeletal physical fitness components, or as a combination of both.

1 Activities including aerobic and/or anaerobic endurance, e.g., intervals in gallop, galloping in sand, trot or gallop of leash with handler riding bike.

2 Activities requiring muscular endurance, strength, power, stability, balance, mobility or agility, such as parkour, drag weight, weight vest during walking, jumping technique exercises, balance training exercises, tricks, static stretching, walking in snow and on uneven surfaces, under water treadmill training, cavaletti.

3 Activities requiring both cardiorespiratory and musculoskeletal components of physical fitness, e.g., hill climbing, running, agility, swimming, canicross, bikejioring, off-leash exercise in the forest, treadmill.

With regards to sport-specific training, 60% (*n* = 953) received at least 3 h of training per week and only a very small portion (*n* = 35, 2%) trained their specific discipline less than once per week (Tables 4, 5).

There was variability in the number of disciplines participated in through the whole cohort, with a range from one to five. Most commonly reported was one discipline (*n* = 767, 48%) and two disciplines (*n* = 541, 34%). Three dogs (2%) were competing in five disciplines, 50 dogs (3%) in four disciplines and 221 dogs (14%) in three disciplines. Of the dogs practicing only one discipline, 38% (*n* = 294) were considered specialized since they were actively training that discipline for ≥10 months per year. Among the agility dogs, 20% (*n* = 84) were specialized, while for the other disciplines, the proportions of specialized dogs were around 10%.

Minimum and maximum total work load per week, i.e., daily physical activity, vigorous physical conditioning exercise and sport-specific training combined, were 0.5 and 49 h per week, respectively. Median total work load was 16.5 h (IQR 9.0) per week in the full cohort and in general there was a higher total work load for dogs in the working trial disciplines (Tables 4, 5).

**Figure 1:**
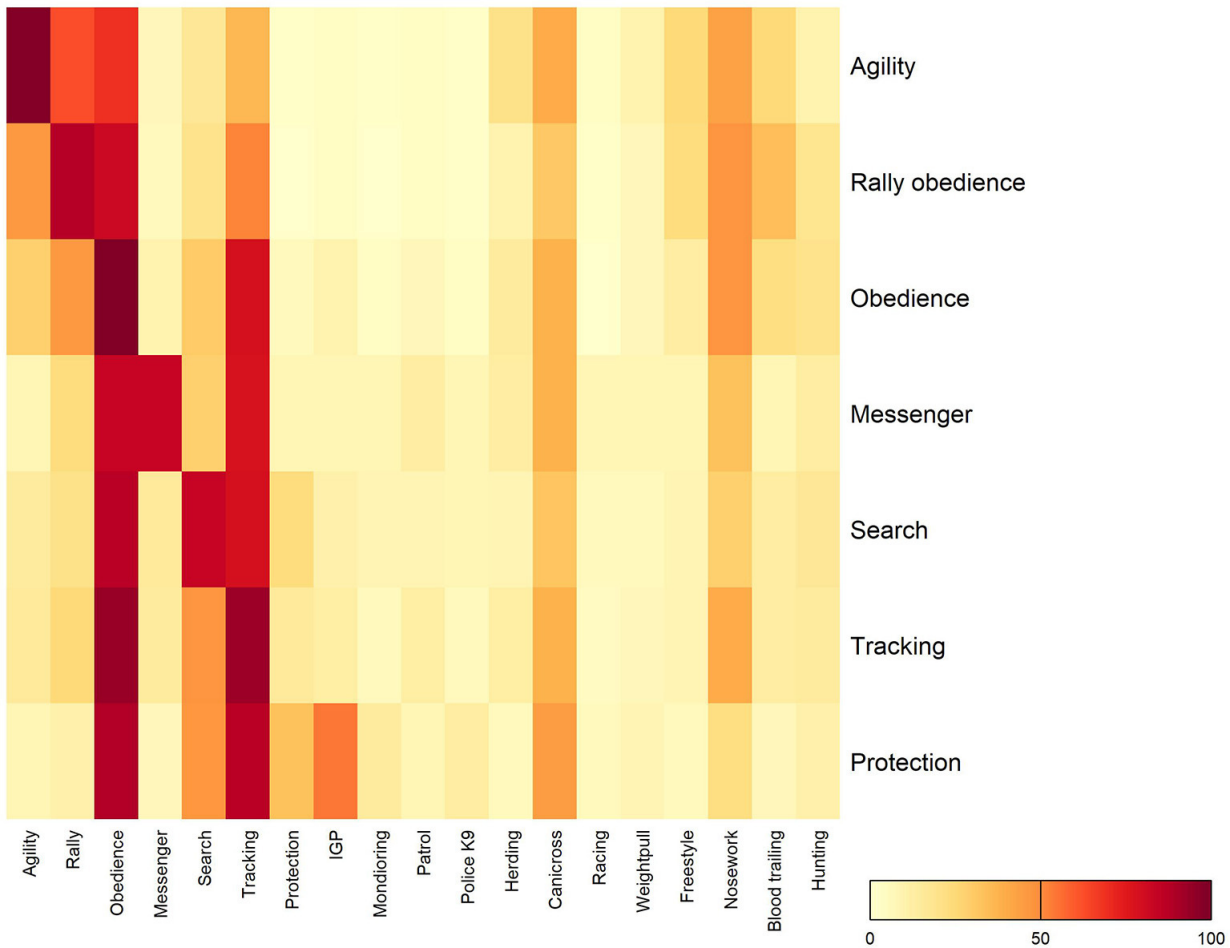
Heat map of participations and interaction in various sporting and working trial disciplines 662 (y-axis) and other physically demanding activities (x-axis) over a year in Swedish sporting 663 and working trial dogs. Gradient proportions of frequency are displayed as colors ranging from yellow (low) to red (high) are shown in the key. IGP, Internationale Gebrauchshunde Prüfungsordnung.

In addition to various sporting and working trial disciplines there were also interactions with other physically demanding activities. Dogs from all disciplines participated to some extent in canicross (Figure 1). Regardless of primary discipline, almost all dogs also participated in obedience, with the exception of agility where only 70% of the agility dogs participated. For tracking, 35% of the agility dogs, 56% of the rally obedience dogs, and 79% of the obedience dogs participated. In contrast, dogs competing in agility, obedience and rally obedience never participated in protection, search, rescue or patrol activities (Figure 1). Participation and gradient proportions of interaction in various disciplines and other physically demanding activities over the year are illustrated in Figure 1. Frequency of selected types of activities (i.e., tracking, search, mushing, obedience, messenger, utility dog protection, Swedish schutzhund, mondioring, protection related to police K9 or guard dog duty, hunting, game tracking, search and rescue, freestyle, patrol, racing, herding, agility, nose work, rally obedience, drag weight/weight pull), over the year is further defined in Table 1.

### Sporting and working dog management

Three quarters of the dogs (*n* = 1202, 76%) participated in warm-up exercises prior to competition and training. Nearly half of the dogs (*n* = 781, 49%) had warm-up sessions lasting 1–10 min. The most common component of physical warm-up was general exercises (*n* = 1400, 89%), and in additionally 45% (*n* = 711) mobility warm-up exercises was performed, and 18% (*n* = 288) were targeting sport-specific activities. Passive methods prior to training and competition, i.e., massage and/or warm blankets, were used in 4% (*n* = 57), and other un-specified warm-up techniques and/or mental preparation were reported in 5% (*n* = 74) of the dogs (Table 6). Data regarding frequency, duration and content of warm-up stratified by working disciplines are presented in Table 7.

**Table 6:** Frequency, duration and content of warm-up activity prior to competition and training in the full cohort of competition dogs (*n* = 1582) and stratified by participation in various disciplines.

	**Full cohort**	**Agility**	**Obedience**	**Rally obedience**	Working^*^
N dogs	1582	430	790	596	847
**Frequency of warm-up before training or competition**					
Never	21 (1.3)	1 (0.2)	8 (1.0)	8 (1.3)	10 (1.2)
Seldom	93 (5.9)	5 (1.2)	50 (6.3)	46 (7.7)	52 (6.1)
Sometimes	266 (16.8)	42 (9.8)	154 (19.5)	116 (19.5)	158 (18.7)
Often	497 (31.4)	108 (25.1)	263 (33.3)	198 (33.2)	280 (33.1)
Always	705 (44.6)	274 (63.7)	315 (39.9)	228 (38.3)	347 (41.0)
**Duration of warm-up session**					
0 min	25 (1.6)	2 (0.5)	12 (1.5)	9 (1.5)	10 (1.2)
1–10 min	781 (49.4)	152 (35.3)	408 (51.6)	302 (50.7)	449 (53.0)
11–20 min	640 (40.5)	223 (51.9)	309 (39.1)	239 (40.1)	323 (38.1)
21–30 min	110 (7.0)	44 (10.2)	45 (5.7)	32 (5.4)	53 (6.3)
31 min or more	26 (1.6)	9 (2.1)	16 (2.0)	14 (2.3)	12 (1.4)
**Content of warm-up**					
General^1^	1400 (88.5)	403 (93.7)	699 (88.5)	519 (87.1)	757 (89.4)
Sport-specific^2^	288 (18.2)	104 (24.2)	139 (17.6)	113 (19)	120 (14.2)
Mobility exercises^3^	711 (44.9)	254 (59.1)	343 (43.4)	290 (48.7)	343 (40.5)
Passive^4^	57 (3.6)	8 (1.9)	34 (4.3)	25 (4.2)	37 (4.4)
Other^5^	74 (4.7)	16 (3.7)	45 (5.7)	37 (6.2)	39 (4.6)

Data are presented in frequencies and proportions (%).

* Working trial disciplines were defined as Swedish Schutzhund, tracking (SWDA), search (SWDA), messenger (SWDA), patrol (SWDA), International Utility Dog trials (tracking, obedience, protection, search and rescue), International Nordic Style, BH/VT, and mondioring. SWDA, Swedish Working Dog Association.

1 Increasing body temperature, e.g., by locomotion in walk and/or trot.

2 Movements and tasks that were to be performed in the upcoming discipline, e.g., heelwork, jumping, bite work, off-leash search for objects, intervals in gallop.

3 Dynamic and/or static stretching in purpose to increase flexibility, e.g., play, tricks, walking in circles or with increased active joint range of motion, locomotion off-leash, short intervals in canter.

4 Massage and/or warm blanket.

5 Unknown physical warm-up and/or mental preparation.

**Table 7:** Frequency, duration and content of warm-up stratified by working trial disciplines.

	**Messenger**	**Protection**	**Search**	**Tracking**
N dogs	33	169	226	667
**Frequency of warm-up before training or competition**				
Never	0 (0.0)	5 (3.0)	1 (0.4)	5 (0.7)
Seldom	2 (6.1)	6 (3.6)	11 (4.9)	41 (6.1)
Sometimes	5 (15.2)	22 (13.0)	44 (19.5)	123 (18.4)
Often	10 (30.3)	52 (30.8)	82 (36.3)	222 (33.3)
Always	16 (48.5)	84 (49.7)	88 (38.9)	276 (41.4)
**Duration of warm-up session**				
0 min	0 (0.0)	5 (3.0)	1 (0.4)	5 (0.7)
1–10 min	18 (54.5)	96 (56.8)	120 (53.1)	348 (52.2)
11–20 min	12 (36.4)	58 (34.3)	93 (41.2)	264 (39.6)
21–30 min	2 (6.1)	10 (5.9)	10 (4.4)	40 (6.0)
31 min or more	1 (3.0)	0 (0.0)	2 (0.9)	10 (1.5)
**Content of warm-up**				
General^1^	31 (93.9)	142 (84)	204 (90.3)	612 (91.8)
Sport-specific^2^	3 (9.1)	35 (20.7)	28 (12.4)	88 (13.2)
Mobility exercises^3^	15 (45.5)	60 (35.5)	99 (43.8)	271 (40.6)
Passive^4^	1 (3.0)	9 (5.3)	9 (4.0)	26 (3.9)
Other^5^	0 (0.0)	11 (6.5)	9 (4.0)	30 (4.5)

Data are presented in frequencies and proportions (%).

1 Increasing body temperature, e.g., by locomotion in walk and/or trot.

2 Movements and tasks that were to be performed in the upcoming discipline, e.g., heeling, jumping, bite work, off-leash search for objects, intervals in gallop.

3 Dynamic and/or static stretching in purpose to increase flexibility, e.g., play, tricks, walking in circles or with increased active joint range of motion, locomotion off-leash, short intervals in canter.

4 Massage and/or warm blanket.

5 Unknown physical warm-up and/or mental preparation.

Main surfaces used for physical activity were natural grass (*n* = 1434, 90%), gravel (*n* = 1209, 76%), snow (*n* = 1147, 72%), forest (*n* = 1481, 934%), and asphalt (*n* = 808, 51%). For sport-specific training the most commonly used surfaces were natural grass (*n* = 1578, 99.5%), gravel (*n* = 1141, 72%), snow (*n* = 1126, 71%), forest (*n* = 1218, 77%), asphalt (*n* = 638, 40%), turf (*n* = 897, 57%), and indoor venue floorings (*n* = 581, 37%) (Table 8). Indoor home flooring and concrete were never used for physical activity. The categories are specified in Supplementary Table 2.

**Table 8:** Main surfaces used for physical activity and sport-specific training in competition dogs (*n* = 1582).

	**Physical** **activity *N* (%)**	**Sport-specific** **training *N* (%)**
Natural grass	1434 (90.4)	1578 (99.5)
Forest	1481 (93.4)	1218 (76.8)
Gravel	1209 (76.2)	1141 (71.9)
Snow	1147 (72.3)	1126 (71.0)
Artificial turf	4 (0.3)	897 (56.6)
Asphalt	808 (50.9)	638 (40.2)
Indoor venue	3 (0.2)	581 (36.6)
Home flooring	0	51 (3.2)
Other water	41 (2.6)	12 (0.8)
Other soft	2 (0.1)	33 (2.1)
Sand	25 (1.6)	10 (0.6)
Other mobile	16 (1.0)	0
Field	13 (0.8)	8 (0.5)
Concrete	0	7 (0.4)
Stone	4 (0.3)	5 (0.3)
Ice	3 (0.2)	1 (0.1)

Data are presented in frequencies and proportions (%).

### Sensitivity analysis

Excluding dogs that did not participate in sport-specific training during the past year or dogs that were deceased did not change the results.

## Discussion

This study provides detailed insight into physical activity patterns and sport-specific training in sporting and working dogs participating in agility, obedience, rally obedience, and working trial disciplines. Important demographic and descriptive data on physical activity and sport-specific patterns are presented together with information on management routines utilized by dog handlers. To our knowledge, no other studies have been conducted on these topics in dogs competing in obedience, rally obedience, and working trial disciplines.

The competition dogs in our cohort were typically 2–6 years of age and out of FCI breed groups 1, 2, and 8. In contrast to recent studies on flyball and agility dogs, where only 28 and 22% of the dogs were sexually intact (37, 40), 77% of the competing dogs in our study were unaltered. One obvious explanation is cultural differences between countries, but there may also be practical and economical explanations influencing decisions whether or not to neuter or spay a competition dog. In Sweden, neutering for reasons other than medical was prohibited by law until 1988. From a breeding perspective there are several arguments against neutering and spaying dogs. For example, the genetic diversity narrows with fewer dogs in the gene pool and potentially important individuals are lost to the gene pool if neutered (64). There are also differences in regulations for participation in sports between countries, making it more or less viable to keep a female dog intact. In Sweden, intact female dogs in heat may participate in various sporting and working disciplines such as agility, bikejoring, canicross, freestyle, international utility dog disciplines, heelwork to music, herding, mondioring, rally obedience, and obedience. In other disciplines, like the SWDA disciplines, bitches in season are not allowed to compete, but the entry fee is refunded if the dog is in heat. SWDA trials were adopted as a tool to evaluate breeding characteristics, e.g., physical and mental capacities, and workability, in working breeds, and incentives to keep females intact were thus needed. National regulations in other countries may, and do, differ with possible effects on the composition of the competing dog population.

As many as 77% of the dogs had an official mental evaluation through participation in behavior and personality tests. The large proportion can be explained by the mandatory requirement for passing a test prior to competing in the Swedish national working trial disciplines. There seems to be a growing interest from Swedish breeders and dog owners in obtaining behavior and personality assessment in their dogs (65). Altogether, undergoing behavior and personality assessments, structural and conformational evaluations, and hip and elbow dysplasia screenings indicate that handlers of sporting and working trial dogs are compliant to breed-specific health screening programs initiated by breed clubs and the Swedish Kennel Club.

Previous studies on physical activity have shown that agility dogs in USA were walked for ≤2 h per week (39) while agility dogs in Finland were walked for 1.5 h per day (34). Our study confirms the longer duration of walks in Nordic agility dogs compared to American. We further extend these studies by reporting physical activity patterns of several additional disciplines. With regards to previously reported time-based levels of activity, sporting and working trial dogs in our sample exhibited moderate to high durations and moderate to vigorous intensities of physical activity (31). However, the level of intensity of the physical activity is difficult to study using self-reported data. To target intensity we designed questions addressing normal gaits of the dogs in order to capture low to moderate and vigorous levels of physical activity, as previously suggested (26, 27). To further separate vigorous intensity from low to moderate, the respondents were provided with a description of vigorous intensity as physical activity resulting in hard panting. This description is in line with a perceived exertion scale for dogs (60). For agility dogs, it has been reported that 49% were walked mostly, or always, on leash (34). One explanation for this could be to prevent the dogs from galloping and implement variability to the time spent in physical activity. Another explanation for using leash could be laws in some countries that require dogs to stay on a leash while in public. There may also be a lack of readily available free areas to be off leash and a need to protect dogs from road traffic accidents. In our study, we found a higher proportion of dogs spending more than half of their time off leash, and almost all dogs preferred the faster gaits, trot or gallop, as self-selected gaits.

We further observe that more than half of the dogs received vigorous physical conditioning exercise for more than 2 h per week and a slightly higher proportion had weekly regular play sessions with other dogs. In general, we did not notice any differences across disciplines with regards to duration and intensity in physical activity patterns. Adding sport-specific training, we observed a higher total work load in hours per week for dogs participating in messenger trials. We note that this is in line with previous findings that large high drive dogs generate more physical activity with their owners (66). One possible explanation to the moderate to high levels of activity with regards to duration of physical activity, could be the law Outdoor Access Right that gives people the right to freely roam the natural property in Sweden. Hence, the opportunity to walk, cycle, ride, ski, and camp on any land, with the exception of private gardens, near a dwelling house or land under cultivation. Another potential explanation for the moderate to high levels of activity in our sample is that the Swedish Animal Welfare Law (67) regulates the management of pet and competition dogs. For example, dogs are not allowed to be held in crates or on leash indoors, and dog owners have to walk their dogs at least every 6 h during the day.

Sport-specific training was typically conducted several times per week or daily, and lasted for at least 3 h every week in 60% of the dogs in the full cohort. Previous studies have presented conflicting information regarding the duration of weekly training in agility dogs (34, 52). Our findings show that in agility, nearly half of the dogs trained for 3 h or more per week, which is a marked increase of sport-specific training when compared to Finnish and American dog populations where the dogs were reported to train 18 min and <2 h per week, respectively (34, 39). There is increased access to indoor training facilities in Sweden lately, which has increased the availability of agility training over all four seasons, and it should be noted that compared to other disciplines, the total work load for the Swedish agility dogs did not differ. Our study further expands the knowledge on physical activity and sport-specific patterns also in obedience, rally obedience, and working trial dogs, which have not been reported previously. We observe that almost all dogs, regardless of major sport discipline, participated in obedience and tracking activities. In comparison, only 24% of Finnish competitive agility dogs participated in additional physically demanding activities (34). Possible explanations for the differences between the studies could be that our present study targeted dog owners active in SWDA, with local clubs traditionally organizing various types of competitions and thus agility handlers with an interest also in other dog sports, while the Finnish study collected data on primarily agility focused handlers with (potentially) less interest in other sports.

We further observe a higher proportion of injured dogs in our study compared to previous reports of 8–42% injured dogs (39, 50, 51, 53, 68), while we found 58% of the dogs ever being injured. Possible explanations could be differences in the definitions of injuries between the studies, whether or not injuries were confirmed by a veterinarian or not, and if the reported injuries were sports-related or if occurred in another context. More research is needed on risk and protective factors associated to injuries in sporting and working trial dogs.

Warm-up and physical conditioning exercise for the dogs were established routines among dog handlers in our study. However, cardiorespiratory conditioning alone was generally performed only occasionally or not at all. Regular warm-up, prior to training or competition, seemed to be especially well established amongst handlers of agility dogs. These results are in line with data previously reported in agility dogs, indicating that the vast majority performed warm-up activities prior to training or competition (34, 54).

Differences across disciplines regarding surface types used for physical activity and sport-specific training were observed in this study. Clearly, outdoor surfaces, e.g., natural grass, forest, gravel, snow, and asphalt, were mainly used for physical activity. Sport-specific training was also practiced outdoors, but indoor facilities, artificial turf, and other indoor venue surfaces were used as well. Field surfaces and possible relationships with sport-related injuries and performance have been extensively evaluated in human and equine science, but is still a severely unexplored topic in canine athletes.

The design of this study entails certain strengths. The survey approach made it possible to reach out to several sport disciplines covered by the main organization, the Swedish Kennel Club. The full cross-sectional data set was collected over a specific period in time and the information about the opportunity to participate in the study could reach all dog handlers with access to internet at the same time. In this way, we obtained large amount of detailed information about physical activity patterns, sport-specific training, sport specialization, and management of sporting and working trial dogs. We further conducted sensitivity analyses to examine the internal validity of our study. There are also limitations in our study. First, participation was anonymous and did not collect any demographic information about the dog handlers, or about their experience as dog handlers or dog trainers. Second, we did not include any variable on functional recovery following the dogs' participation in physical activity and/or sport-specific training. There is also the possibility of recall bias, i.e., a deviation between the self-reported and the true value of the measurement, a problem well known in questionnaire studies. The use of interval categories for self-reported physical activity, used in this study, is one way of achieving more accuracy in the data (41, 60). However, 30–37% of the dogs participating in the present study spent 3 h or more per week in vigorous physical conditioning exercise. In order to fully reflect the actual time spent in vigorous physical conditioning exercise in future studies, the authors recommend to further specify the higher durations into several categories. For example, add 3–4, 4–5, 5–6, and >6 h.

In conclusion, in a cohort of Swedish sport and working trial dogs, we observe physical activity at moderate to high durations at moderate to vigorous intensities. Most dogs received physical conditioning exercise, but not all dogs were warmed up before training and competition. Our study provides veterinary professionals and dog trainers with valuable insights on the physical exposures and management routines of sporting and working trial dogs.

## Data availability statement

The raw data supporting the conclusions of this article will be made available by the authors, upon reasonable request.

## Ethics statement

In accordance with the national animal ethical guidelines provided by the Swedish codes of statutes: SFS 2018:1192 and SJVFS 2019:9, ethical review and approval was not required for this animal study. The participants were informed about the content and purpose of the study, that responding was entirely voluntary, and that all data was anonymous. Respondents provided informed consent for their dogs to participate when they chose to proceed and submit the electronic questionnaire. Written informed consent was obtained from the owners for the participation of their animals in this study.

## Author contributions

All authors listed have made a substantial, direct, and intellectual contribution to the work and approved it for publication.

## Funding

This research was funded by AGRIA and Swedish Kennel Club joint research fund. Grant number N2019-0020. The funder AGRIA was not involved in the study design, collection, analysis, interpretation of data, the writing of this article, or the decision to submit it for publication.

## Conflict of interest

Author AE was employed by Djurkliniken Gefle IVC Evidensia. Authors AH and CK were self-employed at EmpowerPhysio and Veterinär Catarina Kjellerstedt, respectively. The remaining authors declare that the research was conducted in the absence of any commercial or financial relationships that could be construed as a potential conflict of interest.

## Publisher's note

All claims expressed in this article are solely those of the authors and do not necessarily represent those of their affiliated organizations, or those of the publisher, the editors and the reviewers. Any product that may be evaluated in this article, or claim that may be made by its manufacturer, is not guaranteed or endorsed by the publisher.
