# Supplementary material for: Physical activity and sport-specific training patterns in Swedish sporting and working trial dogs—A questionnaire survey
Source: Front Vet Sci. 2022 Nov 1;9:976000. doi: 10.3389/fvets.2022.976000 (PMC9664515; doi:10.3389/fvets.2022.976000)
Supplement: Supplementary file 1 [file Data_Sheet_1.pdf]

Supplementary Material

Supplementary Table 1. Categorization of sports and working trials into groups based on the functional tasks performed by the dogs.

**Category**	**Disciplines included**
Agility	As organized by SWDA
Obedience	As organized by SWDA
Rally obedience	As organized by SWDA
Working dog trials (as organized by SWDA)	Swedish Schutzhund<br>Tracking (SWDA)<br>Search (SWDA)<br>Messenger (SWDA)<br>Patrol (SWDA)<br>International Utility Dog trials (tracking, obedience, protection, search and rescue)<br>International Nordic Style<br>Mondioring<br>Companion dog exam (international examination of obedience exercises, testing in traffic, encounter with other dogs and behavior of the tethered dog)
Messenger	As organized by SWDA
Protection	Swedish Schutzhund<br>IGP protection<br>Mondioring
Search	As organized by SWDA
Tracking	Tracking (SWDA)<br>Tracking (international tracking trials)

IGP; Internationale Gebrauchshunde Prüfungsordnung, SWDA; Swedish Working Dog Association

Supplementary Table 2. Categorization of the main surfaces used for physical activity and sport-specific training.

**Category**	**Surfaces included**
Natural grass	Grass
Turf	Artificial turf
Forest	Woods, forest
Field	Fields, meadow
Gravel	Gravel road, gravel
Sand	Sand, beach
Asphalt	Asphalt
Stone	Rocks, stones
Concrete	Concrete
Snow	Snow
Ice	Ice
Indoor venue	Matting, agility mat, rubber mat
Home flooring	Wooden floors, yoga mat, indoor mat, patio deck, plastic flooring, tiles, laminate flooring, parquet flooring
Other – water	Water
Other – mobile	Treadmill, balance equipment, under water treadmill
Other – soft	Dirt, horse riding surface, wood ships, peat

Supplementary Table 3. Distribution of the breeds of the sporting and working trial dogs (n=1580) in the study. Data are presented in frequencies and proportions (%).

Breed	N(%)
Airedale Terrier	1 (0.1)
American Akita	1 (0.1)
American Cocker Spaniel	1 (0.1)
American Staffordshire Terrier	5 (0.3)
Australian Cattledog	17 (1.1)
Australian Kelpie	76 (4.8)
Australian Shepherd	86 (5.4)
Bearded Collie	7 (0.4)
Belgian Shepherd – Groendal	13 (0.8)
Belgian Shepherd – Laekenois	2 (0.1)
Belgian Shepherd – Malinois	111 (7)
Belgian Shepherd – Tervueren	35 (2.2)
Berger de Beauce	11 (0.7)
Bichon Frise	3 (0.2)
Black Russian Terrier	2 (0.1)
Bohemian Shepherd Dog	4 (0.3)
Bolognese	1 (0.1)
Border Collie	133 (8.4)
Border Terrier	14 (0.9)
Boston Terrier	4 (0.3)
Bouvier des Flandres	3 (0.2)
Boxer	40 (2.5)
Bracco Italiano	1 (0.1)
Briard	15 (0.9)
Cairn Terrier	1 (0.1)
Cavalier King Charles Spaniel	7 (0.4)
Chihuahua	2 (0.1)
Clumber Spaniel	1 (0.1)
Collie, rough	12 (0.8)
Collie, smooth	14 (0.9)
Curly Coated Retriever	1 (0.1)
Dachshund, miniature	1 (0.1)
Dachshund, rough coated	1 (0.1)
Dalmatian	4 (0.3)
Danish Swedish Farmdog	13 (0.8)
Dobermann	19 (1.2)
Dogo Argentino	1 (0.1)
Dutch Schapendoes	6 (0.4)
English Cocker Spaniel	13 (0.8)
English Pointer	1 (0.1)
English Springer Spaniel	14 (0.9)

Eurasier	3 (0.2)
Finnish Spitz	4 (0.3)
Flat Coated Retriever	32 (2)
French Bulldog	2 (0.1)
German Rough Haired Pointer	2 (0.1)
German Shepherd Dog	205 (13)
German Short Haired Pointer	5 (0.3)
Giant Schnauzer, black	22 (1.4)
Giant Schnauzer, pepper and salt	3 (0.2)
Golden Retriever	39 (2.5)
Gos D'Atura Catala	1 (0.1)
Griffon Belge	1 (0.1)
Hollandse Herdershond , short haired	17 (1.1)
Hollandse Herdershond, long haired	3 (0.2)
Hovawart	30 (1.9)
Ibizian Podenco	1 (0.1)
Icelandic Sheepdog	2 (0.1)
Irish Red Setter	1 (0.1)
Irish Soft Coated Wheaten Terrier	7 (0.4)
Irish Terrier	1 (0.1)
Italian Cane Corso	2 (0.1)
Jack Russell Terrier	5 (0.3)
Jämthund	1 (0.1)
Japanese Spitz	3 (0.2)
Keeshond	1 (0.1)
Kromfohrländer	5 (0.3)
Labrador Retriever	67 (4.2)
Lagotto Romagnolo	8 (0.5)
Lancashire Heeler	3 (0.2)
Landseer	1 (0.1)
Lapponian Herder	6 (0.4)
Leonberger	2 (0.1)
Lhasa Apso	1 (0.1)
Long-haired Pyrenean Sheepdog	5 (0.3)
Medium Size Spitz	3 (0.2)
Miniature American Shepherd	3 (0.2)
Miniature Pinscher	4 (0.3)
Miniature Schnauzer	6 (0.4)
Miniature Spitz	1 (0.1)
Mixed breed	46 (2.9)
Mudi	4 (0.3)
Nederlandse Kooikerhondje	9 (0.6)
Norweigan Buhund	1 (0.1)

Nova Scotia Duck Tolling Retriever	30 (1.9)
Papillon	8 (0.5)
Parson Russell Terrier	3 (0.2)
Perro de Agua Español	9 (0.6)
Perro Sin Pleo del Peru Medio	2 (0.1)
Petit Brabacon	2 (0.1)
Pharao Hound	3 (0.2)
Picardy Sheepdog	1 (0.1)
Pinscher	1 (0.1)
Polski Owczarek Nizinny	1 (0.1)
Poodle, giant	6 (0.4)
Poodle, miniature	9 (0.6)
Poodle, standard	21 (1.3)
Portugese Water Dog	3 (0.2)
Pug	1 (0.1)
Pumi	12 (0.8)
Pyrenean Sheepdog – Smooth faced	5 (0.3)
Rhodesian Ridgeback	3 (0.2)
Rottweiler	49 (3.1)
Russkaya Tsvetnaya Bolonka not FCI	1 (0.1)
Saluki	1 (0.1)
Samoyed	1 (0.1)
Schipperke	1 (0.1)
Schnauzer, pepper and salt	2 (0.1)
Shetland Sheepdog	67 (4.2)
Shiba	3 (0.2)
Shih Tzu	1 (0.1)
Stabijhoun	4 (0.3)
Staffordshire Bullterrier	15 (0.9)
Swedish Lapphund	1 (0.1)
Swedish Vallhund	5 (0.3)
Terrier Brasileiro	2 (0.1)
Volpino Italiano	1 (0.1)
Weimaraner	2 (0.1)
Welsh Corgi Cardigan	9 (0.6)
Welsh Corgi Pembroke	2 (0.1)
Welsh Springer Spaniel	4 (0.3)
West Siberian Laika	1 (0.1)
Whippet	6 (0.4)
White Swiss Shepherd Dog	12 (0.8)
Working Kelpie not FCI	4 (0.3)
Yorkshire Terrier	1 (0.1)
